# Impact of environmental factors on aquatic biodiversity in roadside stormwater ponds

**DOI:** 10.1038/s41598-019-42497-z

**Published:** 2019-04-12

**Authors:** Zhenhua Sun, Ekaterina Sokolova, John E. Brittain, Svein Jakob Saltveit, Sebastien Rauch, Sondre Meland

**Affiliations:** 1 Chalmers University of Technology, Architecture and Civil Engineering, Water Environment Technology, 412 58 Gothenburg, Sweden; 2 University of Oslo: Natural History Museum, University of Oslo, PO 1172, Blindern, 0318 Oslo Norway; 3 Norwegian University of Life Sciences, Faculty of Environmental Sciences and Natural Resource Management, PO 5003, 1432 Ås, Norway; 4 Norwegian Institute for Water Research (NIVA), Gaustadalléen 21, 0349 Oslo, Norway

## Abstract

Constructed stormwater ponds mitigate runoff volumes and pollution, and provide other ecosystem services, such as supporting biodiversity, but these services attracted relatively less attention. The impacts of the pollution levels in the water column and sediments, the physical characteristics of ponds, and the presence of amphibians on the macroinvertebrate community composition and biodiversity were explored in twelve stormwater ponds in Norway. Also, the similarities between macroinvertebrate, zooplankton and plant communities were explored. Most of the taxa displayed in the ordination diagram were positively correlated with the pond size and the number of neighbouring ponds, and negatively correlated with the pollution levels in the water column and sediments. However, no statistically significant impacts on the number of taxa and Shannon index were observed. There were low similarities between the macroinvertebrate and zooplankton community compositions as well as between the plant and macroinvertebrate community compositions in the stormwater ponds. We observed a significant positive correlation between the number of plant and of zooplankton taxa, and a weak non-significant positive correlation between the number of plant and of macroinvertebrate taxa. Overall, the explanatory variables had a significant impact on the community composition, but not on the number of taxa nor Shannon index.

## Introduction

Roads are now widespread around most of the world, and people rely on vehicles for transportation in their daily life. However, roads constitute a major source of pollutants to the environment and adjacent ecosystems. The increasing number of vehicles as well as road construction and maintenance increase pollutant loads from non-point sources, leading to potential impairment of the ecological conditions^1^. Examples of the road-related non-point sources of pollutants include brake linings and tires, petrol and diesel combustion products, and asphalt^2^. As result, road runoff contains various pollutants, e.g. metals, polycyclic aromatic hydrocarbons (PAHs), and salts^3,4^, which can potentially affect the aquatic organisms in receiving water bodies^5^. Road runoff typically contain high levels of particles and many pollutants are bound to these particles. Calmano *et al*.^6^ estimated that more than 90% of the metal load in the aquatic system is bound to particles and settles as sediments. Aquatic organisms can be affected by metals through direct contact to epithelial tissues, or through ingestion of food, detritus or sediment particles^7^. Besides particles, chloride also closely linked to the toxicity of road runoff. Road salt, which is a major source of chloride in road runoff during winter, has been proven to have various harmful effects on the flora and fauna of aquatic systems^8,9^. In addition, the pollutants in the sediments can be released into the water column and/or accumulate in plant and animal tissues^10^ by ionic exchange resulting from increased chloride and/or reducing conditions resulting from oxygen depletion.

Stormwater ponds are commonly constructed along roads to reduce peak runoff flows and to prevent pollutants from reaching ground-water or surface waters^11^. Since a significant portion of pollutants entering the ponds is associated with particles, the main treatment process in stormwater ponds is sedimentation, and sediments in stormwater ponds have been proven to act as reservoirs of pollutants^4^. Hence, organisms in the ponds may be exposed to waterborne pollutants during runoff episodes and from pollutants in the sediment, threatening the integrity of the aquatic ecosystem. For example, a substantial kill of amphibian tadpoles, has been observed as a result of input of highly polluted tunnel wash water^12^.

In addition to the pollutants in the water column and sediments, ecological interactions, such as competition and predation, are also a major aspect that affects biodiversity. Competition determines the abundance and location of individuals within the ecosystem^13^. Predation influences both density and composition of prey populations either directly through consumption or indirectly through trait-mediated interactions^14^.

The knowledge on impacts from pollutants in sediments and water column as well as ecological interactions enables a better understanding of the role of stormwater ponds in supporting and maintaining aquatic biodiversity. However, few studies have included a wide range of potential factors into single study. The existing studies on sediments focused on either heavy metals^15–17^ or organic pollutants^4,18^. In addition, few studies^19,20^ have analysed the ecological interactions between different biological communities in stormwater ponds. The present study focused on testing the following hypotheses:

1. The environmental variables affect the macroinvertebrate community composition and biodiversity;

- Increased pond size, age and number of neighbouring ponds increase the macroinvertebrate richness and biodiversity;
- Increased distance to the nearest neighbouring pond and increased traffic density decrease the macroinvertebrate richness and biodiversity;
- Increased pollution levels in the ponds decrease the macroinvertebrate richness and biodiversity;

(2) The presence of amphibians negatively affects the macroinvertebrate community;
(3) The macroinvertebrate community composition affects the zooplankton community composition, and increased number of macroinvertebrate taxa decreases the number of zooplankton taxa;
(4) The plant community composition affects the macroinvertebrate community composition, and increased number of plant taxa increases the number of macroinvertebrate and zooplankton taxa.

## Results

### Pollutant levels in the ponds

**Figure 1:**
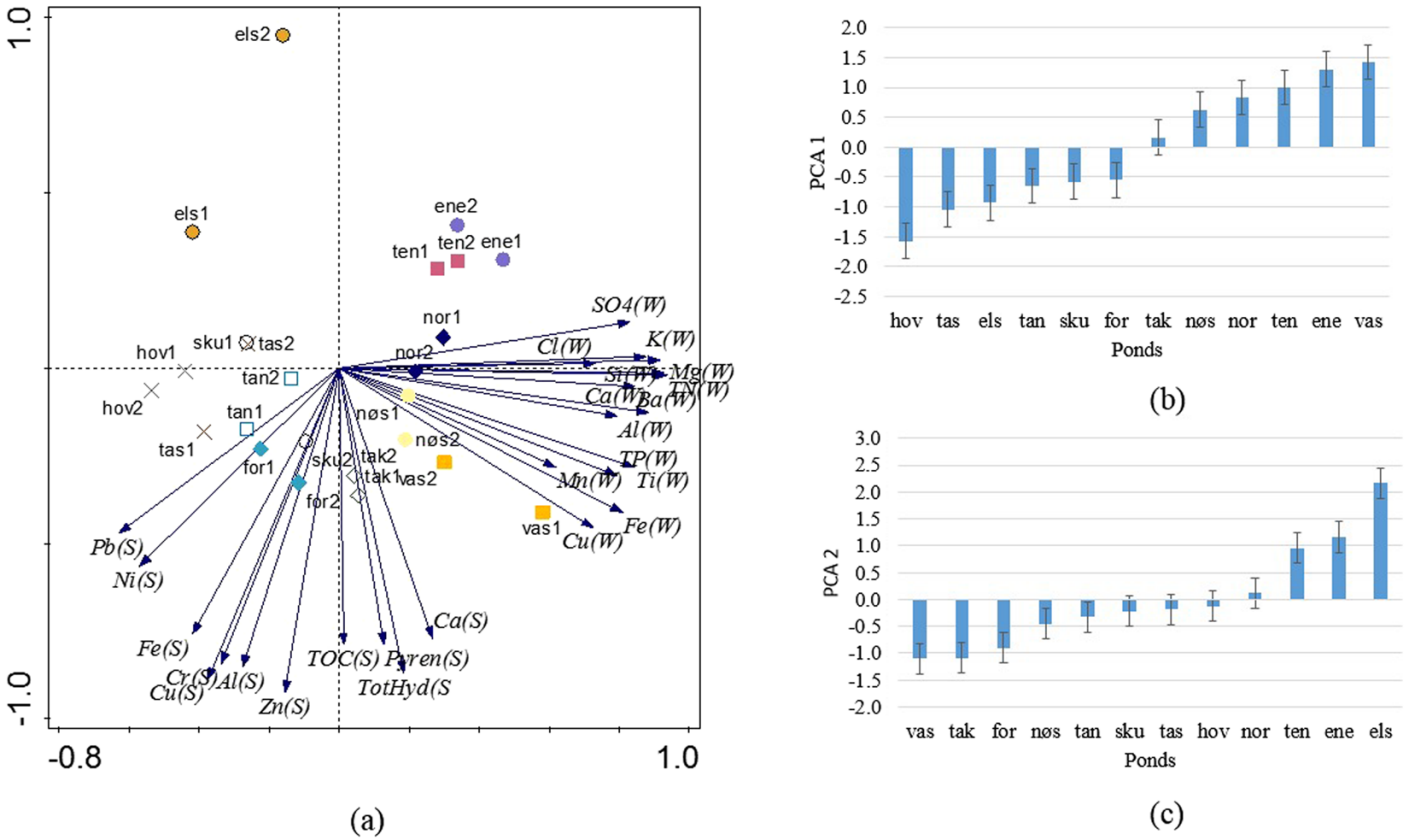
(**a**) Principal components analysis (PCA) for pollutants in the water column and sediments. The following abbreviations are used for the twelve studied ponds: SKU – Skullerud, TAN – Taraldrud North, TAK – Taraldrud crossing, TAS – Taraldrud south, NØS – Nøstvedt, VAS – Vassum, NOR – Nordby, ENE – Enebekk, ELS – Elstadmoen, HOV – Hovinmoen, FOR – Fornebu, TEN – Tenor. W and S in the parentheses represent pollution level in the water column and sediments, respectively. “1” and “2” represent 2013 and 2014. (**b**) Bar chart of mean value of PCA scores extracted from axis 1 for 2013 and 2014 for twelve ponds, increasing sample score indicates increasing pollution level in the water column. (**c**) Bar chart of mean value of PCA scores extracted from axis 2 for 2013 and 2014 for twelve ponds, increasing sample score indicates decreasing pollution level in sediments.

In the principal component analysis (PCA) (Fig. 1a), PCA axes 1 and 2 captured 44% and 30%, respectively, of the total variation of pollutants in the water column and sediments in the twelve studied stormwater ponds. The water quality variables were positively correlated with axis 1, while the sediment variables were negatively correlated with axis 2. Ponds Vassum, Enebekk, Tenor, Nordby, and Nøstvedt had relatively higher concentrations of pollutants in the water column; ponds Vassum, Taraldrud crossing, Fornebu, Taraldrud north, Taraldrud south, Skullerud, and Hovinmoen had relatively higher concentrations of pollutants in the sediments; pond Elstadmoen had lower concentrations of pollutants in the water column and sediments. Ponds Enebekk and Tenor showed high pollution levels in water column, but low pollution levels in sediments. The PCA scores extracted from axes 1 and 2 were used to plot the bar charts (Fig. 1b,c) and were used as proxies for pollution levels in the water column and sediments in further analysis.

### Organisms in the ponds

**Figure 2:**
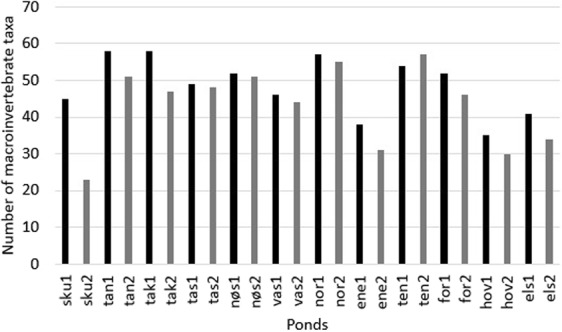
Total number of macroinvertebrate taxa recorded in the twelve studied ponds in 2013 and 2014. “1” and “2” represent 2013 and 2014, respectively. The following abbreviations are used for the twelve studied ponds: SKU - Skullerud, TAN – Taraldrud North, TAK – Taraldrud crossing, TAS – Taraldrud south, NØS – Nøstvedt, VAS – Vassum, NOR – Nordby, ENE – Enebekk, ELS – Elstadmoen, HOV – Hovinmoen, FOR – Fornebu, TEN – Tenor.

A total of 175 macroinvertebrate taxa were sampled and identified, of which four species are red-listed: *Planorbis planorbis* belongs to the data deficient category, *Coenagrion lunulatum* and *Orthetrum cancellatum* belong to the vulnerable category, while *Chaoborus pallidus* belongs to the near threatened category^21^. Most macroinvertebrate taxa (58 taxa) were identified in the ponds Taraldrud North (2013) and Taraldrud crossing (2013), while Skullerud (2014) had the lowest taxa richness (Fig. 2). Four amphibian taxa (one amphibian specimen could not be identified to species) were sampled and identified, i.e. *Rana temporaria*, *Rana arvalis*, *Triturus cristatus*, and *Lissotriton vulgaris*, of which the newt *Triturus cristatus* is red-listed and belongs to the near threatened category^21^.

In addition, 52 zooplankton species were sampled and identified (Fig. S1), of which *Moina macrocopa* is a red-listed zooplankton recorded in Nøstvedt. A total of 57 plant species were recorded along the edge of the ponds, while 21 plant species were found within the ponds (Fig. S1). The ponds Hovinmoen and Elstadmoen exhibited low numbers of macrophyte species (Fig. S1), especially Hovinmoen, in which only three species along the edge of the pond and none within the pond were recorded. This can be explained by the low water level due to leakage, exposing the concrete edges and sides of the ponds, resulting in unfavourable conditions for vegetation.

### Relationships between environmental variables and biological community composition and biodiversity

In the redundancy analysis (RDA), PCA scores extracted from axes 1 and 2 were used to represent pollution levels (Fig. 1a), allowing a reduction from 32 variables to 9 variables: PCA scores from axis 1 representing the pollution level in the water column, PCA scores from axis 2 representing the pollution level in the sediments, presence/absence of frogs, presence/absence of salamander (i.e. newts), pond age, pond size, annual average daily traffic (AADT), number of neighbouring ponds within a radius of 1 km, and distance to the nearest neighbouring pond. To avoid the ordination plots becoming too cluttered, only 25 taxa that were well explained by the first four ordination axes were included (abbreviations of the taxa are explained in Table S3). The PCA result showed that the pollution level in the sediments increased with the decreasing PCA scores. In order to facilitate the understanding of the subsequent RDA, PCA scores that represent pollution level in the sediments were converted to the opposite value, so that in the RDA the pollution level in the sediments increased with the increasing PCA scores.

**Figure 3:**
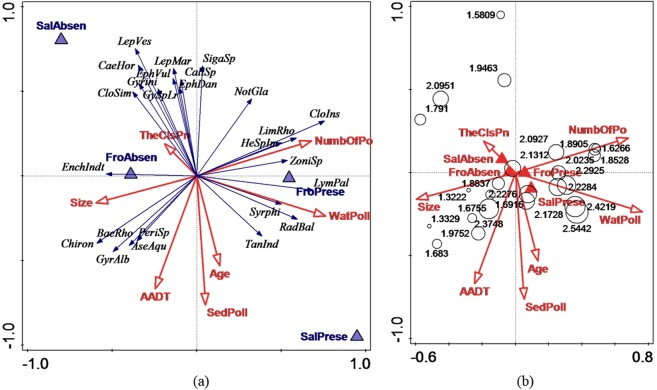
(**a**) Redundancy analysis (RDA) of the relationship between macroinvertebrates and environmental variables as well as amphibians. TheClsPn represents the distance to the nearest pond from the study pond; NumbOfPo represents the number of ponds/water bodies within 1 km; WatPoll and SedPoll represent the pollution levels in the water column and sediments, respectively. FroPrese and FroAbsen represent presence and absence of frogs; SalPrese and SalAbsen represent presence and absence of salamander. (**b**) RDA of the relationship between Shannon indices and environmental variables as well as amphibians. The circles represent Shannon indices.

RDA axes 1 and 2 explained 16% and 14% of the variation in the macroinvertebrate community composition (first axis: p = 0.036; all axes: p = 0.001), respectively. The results (Fig. 3a and Table 1) showed that pollution levels in the sediments and water column, AADT, pond size, distance to the nearest neighbouring pond, and presence/absence of salamander had considerable contribution to the variation in the macroinvertebrate community composition. The pollution level in the water column explained the most (11.5%) of the variation in the macroinvertebrate community composition.

**Table 1:** The marginal effect of each variable on the macroinvertebrate community composition.

Variable	Explains%	p	p (adj)
Pollution level in the water column	11.5	0.002	0.011
Pollution level in the sediments	10.3	0.008	0.018
Number of neighbouring ponds within a radius of 1 km	10.3	0.001	0.011
Annual average daily traffic	10	0.007	0.018
Pond size	9.6	0.004	0.015
Pond age	7.8	0.031	0.057
Presence/Absence of salamander	6.0	0.13	0.171
Distance to the nearest neighbouring pond	5.9	0.14	0.171
Presence/Absence of frog	4.6	0.35	0.362

Most of the displayed taxa (the taxa fitting well to the first four axes) were negatively correlated with the pollution level in the water column, e.g. the mayflies (Ephemeroptera) *Leptophlebia vespertina*, *Leptophlebia marginata*, and *Caenis horaria*, while other taxa, e.g. the snails *Zonitoides* sp. and *Radix balthica*, exhibited a positive correlation. The pollution level in the sediments explained 10.3% of the variation. Most of the displayed taxa were negatively correlated with the pollution level in the sediments, e.g. the mayflies *Ephemera danica*, *Leptophlebia vespertina*, and the caddisfly (Trichoptera) *Limnephilus rhombicus*, while other taxa, e.g. *Radix balthica* and the lake fly Chironomidae, exhibited a positive correlation. The number of neighbouring ponds within a radius of 1 km explained 10.3% of the variation, and some taxa, e.g. *Aeshna cyanea*, Aeshnidae, *Coenagrion pulchellum* and *Radix balthica*, were positively correlated with the number of neighbouring ponds, while taxa such as *Leptophlebia marginata* and *Caenis horaria*, exhibited a negative correlation. Among the 25 dominant taxa displayed in the plots, most taxa exhibited a negative correlation with AADT, with some exceptions, e.g. Chironomidae, Tanypodinae, and *Radix balthica*. Most displayed taxa were positively correlated with the pond size, while taxa, such as the beetle (Coleoptera) *Helophorus* sp. and the snail (Gastropoda) *Lymnaea palustris*, were negatively correlated.

In addition to the species composition, we explored for any differences in number of taxa and Shannon index between the ponds. The non-parametric linear regression using RDA using the ponds as the explanatory variable revealed a significant difference in the number of taxa between the ponds (R^2^_adj_ = 0.63, p = 0.01), but a non-significant difference in the Shannon index between the ponds (R^2^_adj_ = 0.094, p = 0.37). Finally, we explored whether the various environmental variables could explain the observed variation in biodiversity, i.e. number of taxa and Shannon index, between the ponds. The result of the RDA, revealed that the explanatory variables, i.e. the physical characteristics of the ponds, the pollution levels in the water column and sediments, and the presence of amphibians, were not significantly correlated with the number of taxa (R^2^_adj_ = 0.24, p = 0.16) nor the Shannon index (R^2^_adj_ = 0.078, p = 0.36).

### Relationship between different biological communities

**Figure 4:**
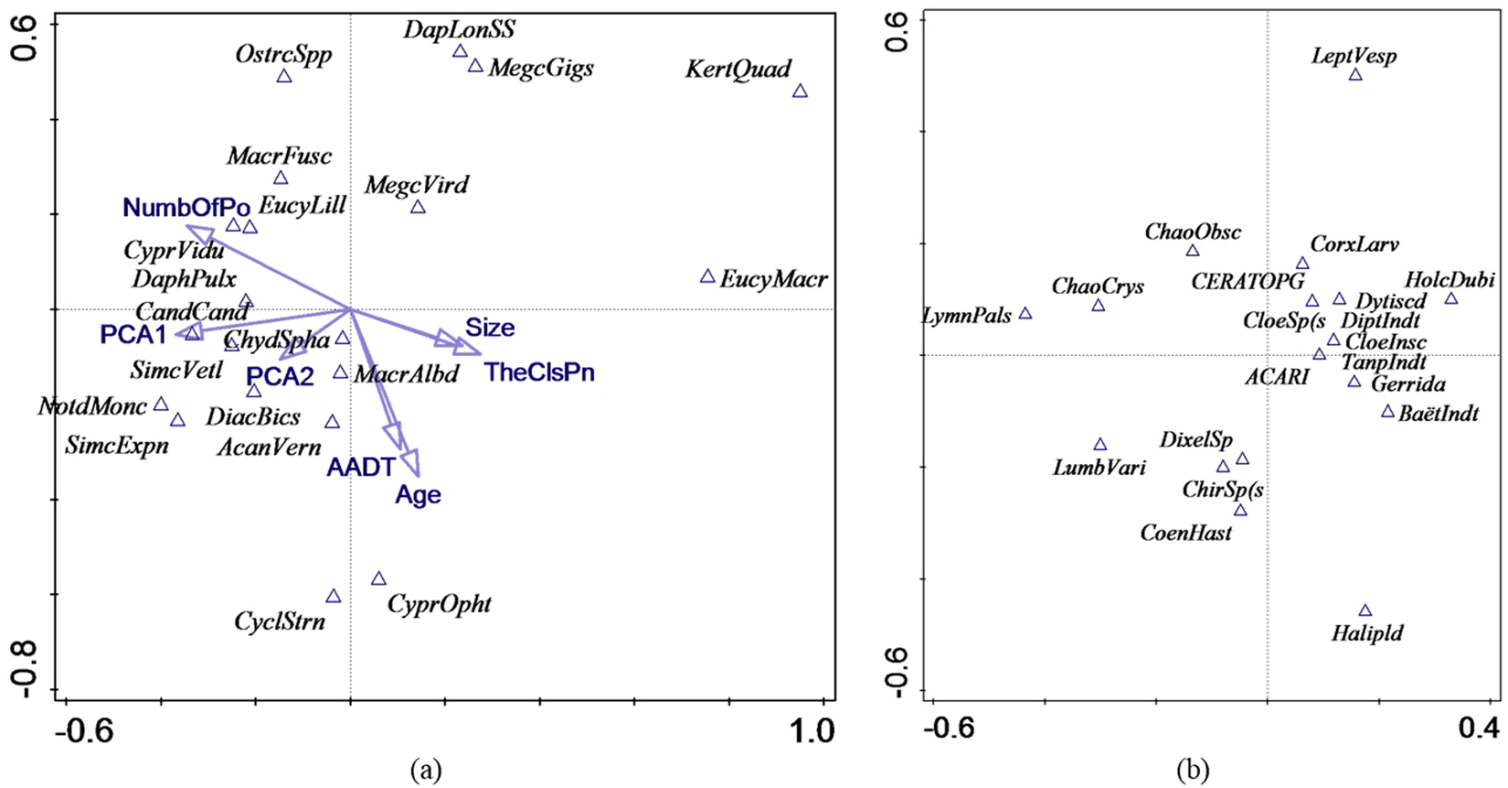
Plot of symmetric co-correspondence analysis with the first two axes and 20 zooplankton (graph a) and 20 macroinvertebrates (graph b) with the largest weight. The environmental variables are projected into graph a.

Co-correspondence analysis (CoCA) was used to explore the relationship between the various biological communities in the twelve ponds, i.e. macroinvertebrate community, zooplankton community and plant community (separated into plants within the ponds and plants along the edge of the ponds). In each graph, only 20 taxa with the largest weight in the analysis were displayed to prevent the ordination plots becoming too cluttered. The ordination diagram of symmetric CoCA for macroinvertebrates and zooplankton is shown in Fig. 4; the species scores of macroinvertebrates and zooplankton were used to display these taxa (abbreviations are explained in Table S4). Cross-correlation was used to examine coherence between the ordination axes. The extent of cross-correlation between the case scores for the two biotic communities for the first four axes was 0.9880, 0.9862, 0.9679 and 0.9785. All axes were significant (first axis: p = 0.048; all axes: p = 0.002). The total inertias of macroinvertebrates and zooplankton were 1.8 and 1.4, respectively, and the total variation captured by CoCA was 0.26. The zooplankton and macroinvertebrates in corresponding positions with respect to the origin in each figure were positively associated, such as the fly *Dixella* sp. and zooplankton *Diacyclops bicuspidatus*, as well as the worm *Lumbriculus variegatus* and zooplankton *Simocephalus vetulus*; the farther these taxa were from the origin, the stronger were their associations.

**Figure 5:**
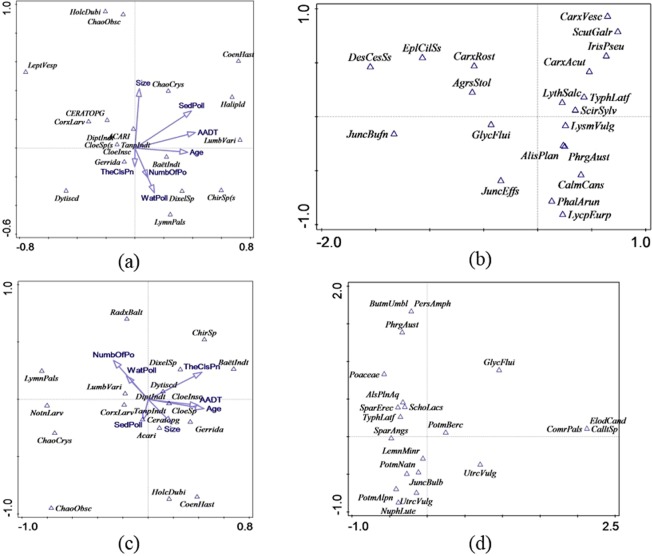
Plot of symmetric co-correspondence analysis with the first two axes and 20 macroinvertebrates (graph a) and 20 plants along the edge of the ponds (graph b) as well as 20 macroinvertebrates (graph c) and 20 plants within the ponds (graph d) with the largest weight. The environmental variables are projected into graphs a and c.

The ordination diagrams of symmetric CoCA for macroinvertebrates and plants are shown in Fig. 5; the species scores of macroinvertebrates and plants were used to display these taxa (abbreviations are explained in Table S5-6). The extent of cross-correlation between the case scores for the macroinvertebrates and plants along the edge of the ponds for the first four axes was 0.9666, 0.9835, 0.9691 and 0.9320, and all axes of CoCA were significant (first axis: p = 0.01; all axes: p = 0.048). The total inertias of macroinvertebrates and plants along the edge of the ponds were 1.8 and 3.4, respectively, and the total variation captured by CoCA was 0.57. Regarding the plant community within the ponds, all axes of CoCA were not significant (first axis: p = 0.176; all axes: p = 0.158), indicating low co-correlation with the macroinvertebrate community.

Non-parametric linear regressions using RDA showed that there was a weak non-significant positive correlation (R^2^_adj_ = 0.11, p = 0.16) between the number of plant taxa and the number of macroinvertebrate taxa in the ponds. On the opposite, there was a significant positive correlation (R^2^_adj_ = 0.42, p = 0.016) between the number of plant taxa and number of zooplankton taxa. Finally, there was a non-significant positive correlation (R^2^_adj_ = 0.15, p = 0.13) between the number of macroinvertebrate taxa and the number of zooplankton taxa.

## Discussion

### Effects of environmental variables on the macroinvertebrate community composition

Regarding pollution level in the water column, the results (Fig. 3) showed positive correlation with some taxa, e.g. the unidentified midge species from the family Tanypodinae, and negative correlation with other taxa, e.g. the mayfly *Leptophlebia vespertina* (Ephemeroptera). Within the taxa that were positively correlated with the pollution level in the water column, some of them are known to be very tolerant to pollution, e.g. Tanypodinae. Dalu *et al*.^10^ also found that Diptera were very tolerant to pollution and normally dominated in polluted areas. Some of the taxa that were positively correlated with the pollution level in the water column were air-breathing organisms, e.g. the large freshwater snails *Radix balthica* and *Lymnaea palustris* from the family Lymnaeidae, which is aquatic pulmonate gastropod^22^, as well as the backswimmer *Notonecta glauca* and the beetle *Helophorus* sp. The fact that these taxa are air-breathing may explain their positive correlation with the pollution in the water column. We also know from previous studies that the oxygen levels in highway stormwater ponds may be hypoxic and even anoxic^23^, especially during wintertime. Such conditions may favour the presence of air-breathing taxa.

The chloride (Cl^−^) concentrations in the current study were above the criteria set by the US EPA – a continuous concentration of 230 mg/L^24^ – in only seven of all 24 collected samples. However, compared with chloride levels typically found in Norwegian lakes (median 1.6 mg/L)^25^, the concentrations of chloride in our study ponds were substantially higher (18–510 mg/L). The elevated concentrations of chloride can result in toxicity due to osmotic stress related to overall ionic strength^26^.

Most of the displayed taxa (i.e. the 25 species that fitted best) were negatively correlated with the pollution level in the sediments, with some exceptions, e.g. *Radix balthica*, Tanypodinae and Chironomidae (Fig. 3). The variables related to the sediments in this study included total organic carbon (TOC), total hydrocarbons, pyrene, and metals. The nature of species exposure and sensitivity to disturbance, as well as species ability to deal with environmental change are determined by various biological and ecological characteristics of each species^27^. The midges, e.g. Tanypodinae and Chironomidae, are normally considered pollution tolerant taxa^10^. Moreover, bioturbation also plays a crucial role in directly and indirectly affecting the toxicity of a sediment associated pollutant to aquatic organisms^28^. Colombo *et al*.^29^ demonstrated that zinc toxicity to the midges significantly decreased due to the presence of *Lumbriculus variegatus*, which changed the sediment geochemistry through digging burrows and depositing a layer of faecal pellets. In this way, zinc concentration decreased in pore water, which is the main exposure pathway to the midges. In our study, the abundance of *Lumbriculus variegatus* was relatively high in some ponds, thus, the toxicity of sediment associated pollutants may be reduced.

Among the taxa that were negatively correlated with the pollution levels in the sediments and water column, many belong to Ephemeroptera and Hemiptera. Several studies have demonstrated that many Ephemeroptera species are sensitive to organic pollution^30,31^ and metals^32^. Therefore, total nitrogen, total phosphorus and metals may result in the negative responses of Ephemeroptera species in our study. On the other hand, Bere *et al*.^16^ found that some Ephemeroptera taxa were highly tolerant to metals, but the Ephemeroptera taxa in their study were only identified to family and, in some cases, class level, hiding species level variability to nutrient concentrations.

Compared to the Norwegian Environmental Quality Standards (EQS)^33^, the concentrations of most toxic metals in the sediments, e.g. lead (Pb), nickel (Ni), and chromium (Cr), were relatively low in this study (Table S2), and most of the samples can be categorized as high/good ecological status (Table S7), except copper (Cu) that was present at elevated concentrations in the ponds Skullerud (2014), Taraldrud north and crossing (2013 and 2014), Taraldrud south (2013), Nøstvedt (2014) and Hovinmoen (2014). Cu typically originates from brake wear. Organic matter in the sediments has a significant effect on the bioaccumulation of metals due to the strong affinity between metals and organic matter^34^. Dissolved and particulate organic matter can act as scavengers for metals, and the scavenged metals may subsequently be incorporated into the bottom sediments^35^. In addition, although TOC itself is not toxic, biodegradation of TOC causes oxygen depletion, leading to suffocation of organisms^36^. Pyrene can result in acute and chronic toxicity, and was used as a proxy for polycyclic aromatic hydrocarbons (PAHs) in the analysis. Compared to the EQS for pyrene, six sediment samples in our study were categorised as “poor quality” that could result in acute toxicity to aquatic organisms. In addition, a recent study of some Norwegian sedimentation ponds has shown that alkylated PAHs may substantially contribute to the total PAH concentrations in these ponds^4^. Therefore, the PAH concentrations in the present study are most likely underestimated^37^.

As an urban drainage system, the stormwater ponds are specifically created to remove pollutants from surface runoff. Even though in the study ponds, the pollution levels were generally moderate, except pyrene, there was an apparent effect on the community composition of the macroinvertebrates. However, there was no evidence that the pollution levels had a negative impact on the biodiversity measured as number of taxa or Shannon index. Hsu *et al*.^38^ also found that except chemical oxygen demand, relations between other chemicals, e.g. total nitrogen, total phosphorus and nitrate, and biotic metrics of macroinvertebrates, i.e. taxa richness and Shannon index, were not clear. Our results show that stormwater ponds could provide suitable habitats for pollution-tolerant taxa, but for taxa that are not tolerant to pollutants, stormwater ponds may not provide additional habitat. Habitat characteristics also act as important factors in shaping the assemblages and compensating the negative effects caused by elevated pollutants. For example, our previous study showed that larger ponds are able to dilute pollutants, thereby creating an environment beneficial for organisms to live^39^. Since stormwater systems may potentially act as ecological traps^40^ and there is no clarity on the impact of pollution levels on the biodiversity, more research is needed on the multifunctionality of stormwater ponds.

Most displayed taxa were positively correlated with the number of ponds (Fig. 3), and this was also demonstrated by our previous study^39^. This is mainly because higher connectivity between ponds facilitates the mobility of invertebrates between ponds, thereby contributing to higher biodiversity. Several aquatic macroinvertebrates have a terrestrial adult stage and require surface water to complete larval stages, e.g. Odonata. Therefore, compared with the network of ponds or “pondscape” as a whole, a single pond may be less important in the ecological value^41^. In our study, several taxa from Odonata were positively correlated with the number of ponds, e.g. *Aeshna cyanea*, Aeshnidae, and *Coenagrion pulchellum*. Some taxa, e.g. *Coenagrion hastulatum*, *Aeshna juncea*, and *Lestes sponsa*, were less dependent on having many ponds nearby. However, the taxa that did not show positive correlation with the number of ponds were positively associated with the pond size and vice versa.

The results of RDA for pond size are also in agreement with our previous study^39^, in which most displayed taxa were positively correlated with pond size, e.g. *Aeshna juncea*, *Coenagrion* sp. and the mayfly *Caenis horaria*. However, a considerable number of displayed taxa exhibited a negative correlation with pond size as well, e.g. *Helophorus* sp., *Lymnaea palustris* and the clam (Sphaeriidae) *Sphaerium* sp. Similar results were obtained by Oertli *et al*.^42^ who demonstrated that there were limitations of species-area relationship in its application to ponds, and that this relationship was apparent for Odonata, but not relevant for Sphaeriidae, Coleoptera and Gastropoda, for which a set of ponds with small size is more favourable than a single large pond of the same size. In addition, since it is impossible to analyse each variable separately due to complex interactions, other variables, e.g. water quality, may play a prevailing role, making the species-area relationship less important and yielding contradictory results for some biological communities. For example, in a study by Søndergaard *et al*.^43^, the relationship with the pond area was weak for macroinvertebrates but strong for submerged macrophytes.

Compared with other variables, pond age had a relatively low impact on the biological community composition in our study. The results showed that more taxa were present in younger ponds (Fig. 3). Scher *et al*.^44^ suggested that older ponds support greater species richness. On the other hand, Gee *et al*.^45^ demonstrated that pond age did not significantly contribute to the number of macroinvertebrate taxa. In our study, there may be uncertainties related to pond age, since maintenance, including removal of sediment and vegetation, may have an effect on the fauna.

In contrast to the apparent and significant impact of the explanatory variables on the community composition, the explanatory variables did not have a significant impact on the biodiversity, i.e. the number of taxa and Shannon index, highlighting the need for more research.

It is important to mention that fish exists in the pond Skullerud. Fish are normally not present in these types of ponds, as they have no direct links to other upstream water bodies. However, the pond Skullerud is very close to the River Ljanselva, and fish has gained access to the pond during high flooding events. Hence, the presence of fish may have influenced the macroinvertebrate and amphibian communities in this pond, as shown in other studies^46,47^.

### Relationship between different biological communities

Although the cross-correlation value shows that each of the CoCA axes obtained for the two communities was almost perfectly correlated, only a small part of the total variation in these two communities was captured by CoCA as co-variation. The results also showed that a large proportion of macroinvertebrate and zooplankton taxa as well as macroinvertebrates and the plants within and along the edge of the ponds were poorly associated, judging by their placements along the axes, suggesting that the similarity was not high.

Macroinvertebrates are expected to co-correlate with zooplankton, since predatory macroinvertebrates, such as odonates, have been demonstrated as effective predators of zooplankton, and the existence of predators normally changes the composition of the zooplankton community^48^. However, in our study, both the number of all macroinvertebrates and the number of odonates showed a non-significant correlation with the number of zooplankton taxa (R^2^_adj_ = 0.012, p = 0.31). Tolonen *et al*.^49^ also found that no congruence existed in the species richness or evenness between macroinvertebrates and zooplankton.

Several studies have found that macroinvertebrate diversity was positively correlated with plant cover^38,50,51^ due to such factors as food availability and shelter from predators. However, the relationships with plant species composition are much less recognized. In the current study, plant species composition did not emerge as the good predictor for studied macroinvertebrate groups. However, the number of plant taxa appeared to be a good predictor for the number of zooplankton taxa, in which the number of zooplankton taxa increased with the increase in the number of plant taxa.

## Conclusion

Below we summarise the findings based on the hypotheses outlined in the introduction:

1. The analysis of the impact of the environmental variables on the macroinvertebrate community composition in constructed stormwater ponds showed that most of the taxa displayed in the ordination diagram were positively correlated with the pond size and the number of ponds within a radius of 1 km, and negatively correlated with AADT and the pollution levels in the water column and sediments. However, the analysis of the impact of the environmental variables on biodiversity, measured as the number of taxa and Shannon index, did not show any statistically significant impacts. Hence, stormwater ponds could provide suitable habitats for taxa that are moderately to strongly tolerant to pollutants.
2. The analysis of the impact of the presence of amphibians on the macroinvertebrate community composition and biodiversity, measured as the number of taxa and Shannon index, did not show any statistically significant impacts.
3. There was a low similarity between the macroinvertebrate community composition and the zooplankton community composition. The relationship between the number of macroinvertebrate taxa and the number of zooplankton taxa in the ponds was not significant.
4. There was a low similarity between the plant community composition and the macroinvertebrate community composition and a weak non-significant positive correlation between the number of plant taxa and the number of macroinvertebrate taxa in the ponds. There was a significant positive correlation between the number of plant taxa and the number of zooplankton taxa in the ponds.

In these moderately (except pyrene) polluted stormwater ponds, we observed an apparent negative effect of pollution levels on the macroinvertebrate community composition, but not on the biodiversity measured as the number of taxa or Shannon index. Therefore, more research is needed to determine to which extent stormwater ponds for road runoff can support biodiversity. In addition, further studies with quantitative biotic data, e.g. abundance of plant and zooplankton species, may provide more information regarding interrelationships between the different biological communities.

## Materials and Methods

### Study area

**Figure 6:**
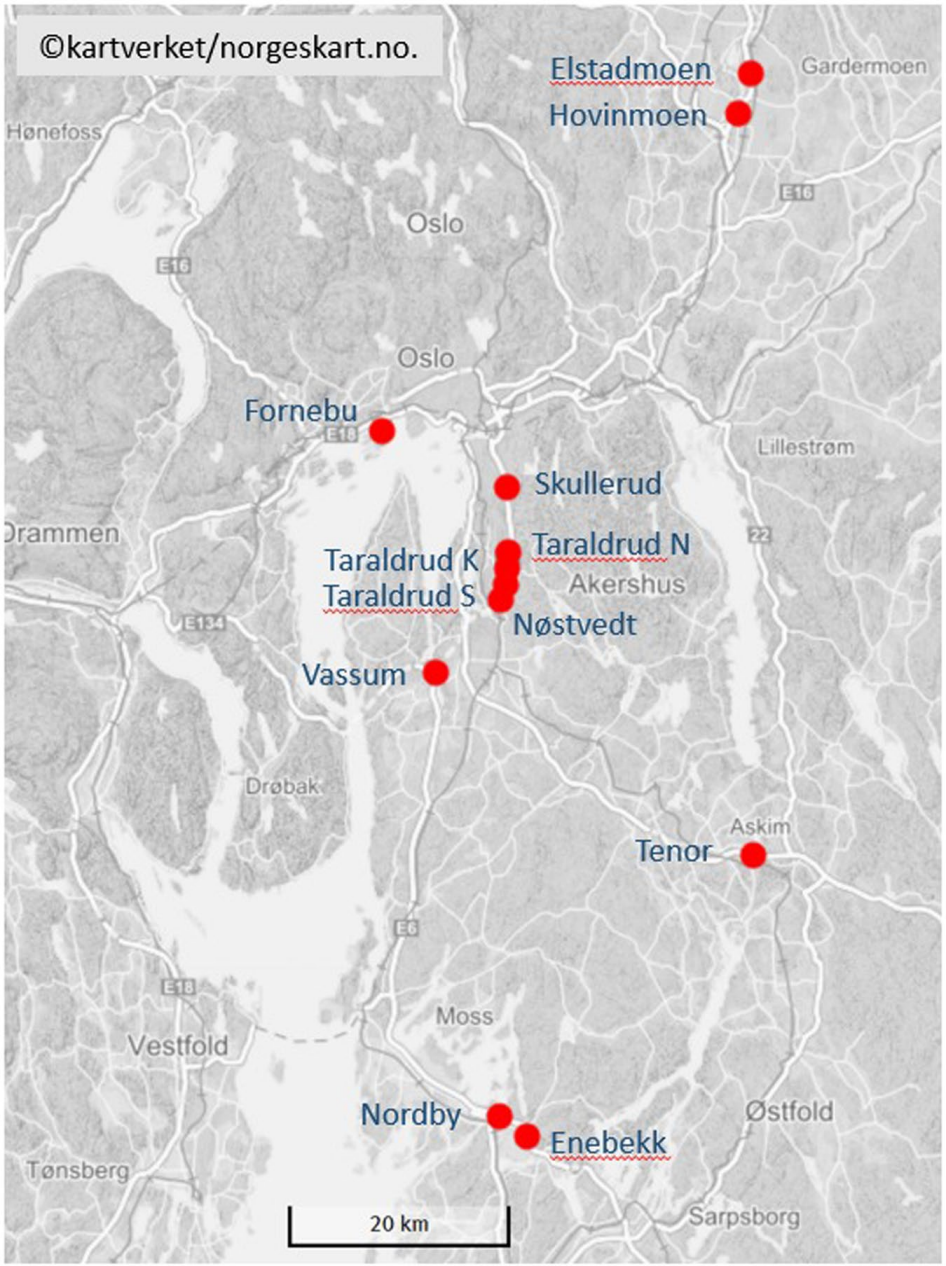
Location of all the studied stormwater ponds (red dots) in the counties of Oslo, Akershus and Østfold. The ponds are: ELS – Elstadmoen, HOV – Hovinmoen, FOR – Fornebu, SKU – Skullerud, TAN – Taraldrud north, TAK – Taraldrud crossing, TAS – Taraldrud south, NØS – Nøstvedt, VAS – Vassum, TEN – Tenor, NOR – Nordby, and ENE – Enebekk. Map is based on ref.^52^.

Twelve stormwater ponds receiving road runoff were explored in this study (Fig. 6). The map was taken from the Norwegian Mapping Authority’s free products^52^. Eight of these ponds were previously studied by Sun *et al*.^39^. In order to increase the geographical range of ponds and the range of pond age, four new ponds were included in the present study. Except for one pond, Fornebu (new) located in an urban area, the ponds are situated along major highways E6 and E18 in the counties of Oslo, Akershus and Østfold in southern Norway.

### Sediment and water quality sampling and analysis

Sediment samples were collected in April in both 2013 and 2014, respectively; the top-layer sediments were taken close to the inlet with a spade and stored in 1 L glass bottles. The spatial distribution of pollutants within a stormwater pond may vary, but highest pollutant concentrations are typically found at the inlet^53^. Eleven sediment variables were analysed in this study, i.e. TOC, total hydrocarbons, US EPA 16 PAHs, aluminium (Al), calcium (Ca), Cr, Cu, iron (Fe), Ni, Pb, and zinc (Zn). The PAH compound pyrene was included in the statistical analysis and used as a proxy for PAH pollution since it was quantified in all samples; other PAH compounds were below the limit of quantification (LOQ) in more than 15% of the total number of samples.

Water samples were collected once in 2013 (April) and three times in 2014 (April, June and August) close to the inlet of the ponds. April, when samples were collected in both 2013 and 2014, is the period with the highest road runoff and concentration of pollutants due to spring snowmelt. Fourteen water quality variables were analysed in this study. Samples for total metal concentration analysis (Al, Ca, Cu, Fe, barium (Ba), potassium (K), magnesium (Mg), manganese (Mn), silicon (Si) and titanium (Ti)) were collected in 125 mL acid washed polyethylene (PE)-bottle, while samples for Cl^−^, total nitrogen (TN), total phosphorus (TP) and sulphate (SO_4_^2−^) analysis were collected in 125 mL PE-bottle. The samples, both water and sediment, were shipped to the laboratory normally the day after sampling. All chemical analysis performed by Rambøll Analytics Laboratories Finland. Conductivity, pH and temperature were measured using handheld probes.

### Physical variables

The data for the physical variables were collected either from digital maps (Norwegian Mapping Authority) or directly from the Norwegian Public Roads Administration (NPRA) (Table 2).

**Table 2:** Physical variables for the studied stormwater ponds.

Ponds	Constructed	Size (m^2^)^a^	Ponds^b^	Distance (m)^c^	AADT^d^
Skullerud (SKU)	1998/1999	910	1	980	66500
Taraldrud north (TAN)	2004	780	3	450	42900
Taraldrud crossing (TAK)	2004	1400	6	120	42200
Taraldrud south (TAS)	2004	474	4	130	42200
Nøstvedt (NØS)	2009	340	3	15	35500
Vassum (VAS)	2000	363	5	30	41000
Nordby (NOR)	2004/2005	89	8	600	22735
Enebekk (ENE)	2004/2005	132	5	587	23837
Tenor (TEN)	2007	480	2	56	12000
Fornebu (FOR)	2002	480	3	203	25000
Hovinmoen (HOV)	2007/2008	422	6	257	19000
Elstadmoen (ELS)	2007/2008	741	2	435	19000

^a^Pond surface area.

^b^Number of neighbouring ponds within a radius of 1 km.

^c^Distance to the nearest neighbouring pond.

^d^Annual Average Daily Traffic.

### Biological sampling and analysis

Macroinvertebrates and amphibian samples were collected four times (April, June, August and October) in both 2013 and 2014, respectively. Traps and a kick net with 30 × 30 cm opening and mesh size of 0.45 mm were used. Large amount of detritus, plant remains, etc. that would severely clog the net is challenging when sampling such habitats. Hence, kick sampling with five sweeps was used when there were small stones on the bottom. When the bottom material was not stony, five sweeps were taken through the water and aquatic vegetation when present at approximately 50 cm depth. In total, we sampled at three sites within each pond, close to the inlet and twice on either side of the main basin. Although five sweeps seem limited, we consider that with our sampling at multiple sites in the ponds and over a period of two years, a true picture of the macroinvertebrate diversity and their relative abundance can be obtained. Care should however be taken when comparing these results with others who may have obtained different sampling strategy.

Traps made of 1.5 L transparent plastic bottles were also used to collect samples from either side of the main pond^39^. The plastic bottles were cut in two parts, and the bottleneck that forms the spout was turned around and placed inside the bottle and attached with transparent tape. Two traps were put into the main pond at approximately the same places as the kick samples were taken; the traps were left for 1–4 days, according to time of the year. Amphibians were sampled together with the benthic fauna, and mostly were caught in the plastic bottle traps (mostly newts), but some (mostly tadpoles of frogs) were also recorded in the net samples together with benthic macroinvertebrates. In addition, egg clusters were observed in field during the spring survey. The recordings of amphibians are semi-quantitative. The samples, except larger specimens such as amphibians, were preserved in 70% ethanol. Organisms were sorted in the laboratory and identified to species level when possible. Nilsson^54^ was used to identify benthic macroinvertebrates to the lowest possible taxonomic level (in most cases, species), and several of the Diptera were identified only to subfamily.

Zooplankton was analysed once in 2013 in the kick and sweep net samples, as well as separate plankton net hauls (mesh 90 µm). Organisms were sorted in the laboratory and identified to the lowest possible taxonomic level. Flössner^55^ and Flössner^56^ were used to identify Cladocera; Sars^57^ and Einsle^58^ for Copepoda; Henderson^59^, Lindholm^60–62^ for Ostracoda and Pontin^63^ for Rotifera.

All identified plants were macrophytes, and most of the plant species were recorded by wading. The abundance/dominance of each species was estimated on a scale 1–3: 1 represents <5% cover (uncommon/rare); 2 represents 5–50% cover (common) and 3 represents >50% cover (abundant/dominant).

### Statistical analysis

#### Sediment and water quality analysis

PCA was used to compare pollution levels in the sediments and water column among different ponds, as well as to reduce the number of variables used in the subsequent constrained analysis, RDA. The sediment and water quality variables of 2013 and 2014 were analysed using PCA, and PCA scores were extracted to represent pollution levels in the water column and sediments. If too many explanatory variables are included in the dataset, the risk of overfitting the RDA model is high (further description of RDA in the next chapter). Therefore, the extracted PCA scores were used in the subsequent RDA. In addition, the mean value of PCA scores of 2013 and 2014 was used to display the differences in pollution levels between the different ponds. The data were log(x + 1) transformed prior to PCA to reduce the skewness and improve the normality of the data.

#### Community analysis

The abundance of macroinvertebrates was used to evaluate the relationship between the macroinvertebrate community composition and the environmental variables, including physical, water quality and sediment variables. The presence/absence of amphibians was used as an explanatory variable to check the relationship between macroinvertebrates and amphibians. Since pH value was relatively constant in our study, it was excluded from the analysis. Conductivity was also excluded, since it was highly correlated with Cl^−^. In each pond, the four sampling campaigns from each year were pooled/aggregated. This was primarily done to have proper comparison with the environmental variables. Secondly, seasonal variation was accounted for, and some of the seasonal variability was removed per year through merging of four samples. The data were log(x + 1) transformed prior to the analyses. The relationship between the variation in the macroinvertebrate community and the environmental variables was evaluated using RDA. In the RDA, Monte Carlo permutation tests (999 permutations, p < 0.05) were used to determine the statistical significance. In addition, the taxa samples were Hellinger transformed^64^ prior to the RDA. Hellinger transformation gives low weights to rare species^65^ to compensate the disadvantage of RDA that is not appropriate with community composition data containing many zeros. The formula of Hellinger transformation is:

1 $${D}_{Hellinger}({x}_{1},{x}_{2})=\sqrt{{\sum _{j=1}^{p}[\sqrt{\frac{{y}_{1j}}{{y}_{1+}}}-\sqrt{\frac{{y}_{2j}}{{y}_{2+}}}]}^{2}}$$

The Shannon index (H’) was used as an ecological indicator with respect to biodiversity^66^ following the equation:

2 $$H^{\prime} =-\sum _{i=1}^{s}{p}_{i}ln{p}_{i}$$

where S is the number of taxa and p_i_ is the relative abundance of each taxon.

A non-parametric multiple linear regression by RDA was applied using the number of taxa and Shannon index as response variable (ran separately) to check whether the biodiversity, measured as Shannon index and the number of taxa, were linked to any of the explanatory variables. Furthermore, two separate RDAs were used to explore whether there were any differences in number of taxa and Shannon index between the ponds. In these two analyses ponds were used as categorical explanatory variable.

In order to explore the similarities between two biotic communities, the ordination method symmetric CoCA^67^ was used. This analysis allows to directly compare two biotic communities by maximizing the covariance between weighted averaged species scores of one community with the weighted averaged species scores of the other community^67^. Prior to CoCA, DCA was performed for each biological community. Four biotic communities were examined in this study: macroinvertebrates, zooplankton, and plants within and along the edge of the ponds. The relative abundance of each community, in which “1” represents “uncommon/rare”, “2” represents “common”, and “3” represents “dominant”, was used for the analysis. The environmental variables, including physical, water quality and sediment variables, were used as supplementary variables so that the compositional covariation in different biotic communities can be interpreted with environmental variables. The displayed taxa in each graph were limited by the weight, which is sum of values over data table column or row for the response variables and cases, and 20 macroinvertebrates with the largest weight were selected. Furthermore, non-parametric linear regression was done based on the number of plant taxa, the number of zooplankton taxa and the number of macroinvertebrate taxa using RDA to check whether the diversity of plant community influences on the diversity of macroinvertebrate and zooplankton community and whether the diversity of macroinvertebrate community has influence on the diversity of zooplankton community. The number of plant taxa used in this analysis was the sum of plant taxa within and along the edge of ponds.

The CANOCO5 software (Micro-computer Power) was used for multivariate statistical analysis.

#### Statement

The sampling of macroinvertebrates was approved by the Norwegian Public Roads Administration, which was interested in documenting biodiversity in stormwater ponds. The collection and identification were carried out by an independent scientific institution: the Natural History Museum, University of Oslo. An ethics approval for experiments on live invertebrates is not relevant to our study because no experiments were carried out on invertebrates. Although four amphibian taxa are included, the amphibians were caught during macroinvertebrates sampling. Amphibians were identified and counted before being released back into the water.

## Supplementary information

Supplementary information

## Data Availability

Datasets are available from the authors upon request.
