# Supplementary material for: Impact of environmental factors on aquatic biodiversity in roadside stormwater ponds
Source: Sci Rep. 2019 Apr 12;9:5994. doi: 10.1038/s41598-019-42497-z (PMC6461623; doi:10.1038/s41598-019-42497-z)
Supplement: Supplementary file 1 — Supplementary information [file 41598_2019_42497_MOESM1_ESM.pdf]

Supplementary Information

Impact of environmental factors on aquatic biodiversity in roadside stormwater ponds

Zhenhua Sun[a,*], Ekaterina Sokolova[a], John E. Brittain[b], Svein Jakob Saltveit[b], Sebastien Rauch[a], Sondre Meland[c,d]

[a] Chalmers University of Technology, Architecture and Civil Engineering, Water Environment Technology, 412 96 Gothenburg, Sweden

*Corresponding author: zhenhua.sun@chalmers.se, +46317721984

[b] University of Oslo: Natural History Museum, University of Oslo, PO 1172 Blindern 0318 Oslo, Norway

[c] Norwegian University of Life Sciences, Faculty of Environmental Sciences and Natural Resource Management, PO 5003, 1432 Ås, Norway

[d] Norwegian Institute for Water Research (NIVA), Gaustadalléen 21, 0349 Oslo, Norway

Table S1.1. Concentrations of organic compounds in the sediments of the twelve studied ponds. “1” and “2” represent 2013 and 2014, respectively.

	Anthracene (mg/kg dw)	Acenaphthene (mg/kg dw)	Acenaphthylene (mg/kg dw)	Benzo(a)anthracene (mg/kg dw)	Benzo(a)pyrene (mg/kg dw)	Benzo(b)fluoranthene (mg/kg dw)
sku1	<LOQ	<LOQ	<LOQ	0.03	0.03	0.06
sku2	<LOQ	<LOQ	<LOQ	<LOQ	<LOQ	<LOQ
tan1	0.05	<LOQ	<LOQ	0.11	0.11	0.16
tan2	<LOQ	<LOQ	<LOQ	<LOQ	<LOQ	<LOQ
tak1	1.2	<LOQ	<LOQ	0.09	0.1	0.15
tak2	<LOQ	<LOQ	<LOQ	<LOQ	<LOQ	<LOQ
tas1	<LOQ	<LOQ	<LOQ	0.1	0.12	0.15
tas2	<LOQ	<LOQ	<LOQ	0.1	0.12	0.12
nøs1	<LOQ	<LOQ	<LOQ	<LOQ	<LOQ	0.06
nøs2	<LOQ	<LOQ	<LOQ	<LOQ	0.11	0.15
vas1	0.16	<LOQ	<LOQ	0.16	0.14	0.2
vas2	<LOQ	<LOQ	<LOQ	<LOQ	<LOQ	<LOQ
nor1	<LOQ	<LOQ	<LOQ	<LOQ	0.02	0.04
nor2	<LOQ	<LOQ	<LOQ	<LOQ	<LOQ	<LOQ
ene1	<LOQ	<LOQ	<LOQ	<LOQ	<LOQ	0.03
ene2	<LOQ	<LOQ	<LOQ	<LOQ	<LOQ	0.02
ten1	<LOQ	<LOQ	<LOQ	<LOQ	<LOQ	<LOQ
ten2	<LOQ	<LOQ	<LOQ	<LOQ	<LOQ	<LOQ
for1	0.05	<LOQ	0.03	0.14	0.17	0.21
for2	<LOQ	<LOQ	<LOQ	<LOQ	<LOQ	<LOQ
hov1	<LOQ	<LOQ	<LOQ	<LOQ	<LOQ	<LOQ
hov2	<LOQ	<LOQ	<LOQ	<LOQ	<LOQ	<LOQ
els1	<LOQ	<LOQ	<LOQ	<LOQ	<LOQ	<LOQ
els2	<LOQ	<LOQ	<LOQ	<LOQ	<LOQ	<LOQ

Table S1.2. Concentrations of organic compounds in the sediments of the twelve studied ponds. “1” and “2” represent 2013 and 2014, respectively.

	Benzo(g,h,i)perylene (mg/kg dw)	Benzo(k)fluoranthene (mg/kg dw)	Dibenzo(a,h)anthracene (mg/kg dw)	Phenanthrene (mg/kg dw)	Fluoranthene (mg/kg dw)	Fluorene (mg/kg dw)
sku1	0.1	<LOQ	<LOQ	0.14	0.21	0.03
sku2	0.37	<LOQ	<LOQ	<LOQ	0.24	<LOQ
tan1	0.32	0.06	<LOQ	0.45	0.65	0.09
tan2	0.31	<LOQ	<LOQ	0.25	0.35	<LOQ
tak1	0.79	<LOQ	<LOQ	2.9	0.88	<LOQ
tak2	<LOQ	<LOQ	<LOQ	<LOQ	<LOQ	<LOQ
tas1	0.36	0.05	<LOQ	0.18	0.39	<LOQ
tas2	0.15	0.05	<LOQ	0.08	0.2	<LOQ
nøs1	0.11	<LOQ	<LOQ	0.15	0.22	<LOQ
nøs2	0.46	<LOQ	<LOQ	0.5	0.59	<LOQ
vas1	0.51	<LOQ	<LOQ	1.2	1	<LOQ
vas2	0.57	<LOQ	<LOQ	0.78	<LOQ	<LOQ
nor1	0.08	<LOQ	<LOQ	0.04	0.09	<LOQ
nor2	0.08	0.025	0.025	0.06	0.11	<LOQ
ene1	0.05	<LOQ	<LOQ	<LOQ	0.04	<LOQ
ene2	0.04	<LOQ	<LOQ	<LOQ	0.03	<LOQ
ten1	0.03	<LOQ	<LOQ	<LOQ	0.03	<LOQ
ten2	0.05	<LOQ	<LOQ	<LOQ	0.03	<LOQ
for1	0.33	0.08	0.03	0.17	0.34	0.09
for2	<LOQ	<LOQ	<LOQ	0.83	0.86	<LOQ
hov1	<LOQ	<LOQ	<LOQ	<LOQ	<LOQ	<LOQ
hov2	<LOQ	<LOQ	<LOQ	<LOQ	<LOQ	<LOQ
els1	<LOQ	<LOQ	<LOQ	<LOQ	0.07	<LOQ
els2	<LOQ	<LOQ	<LOQ	<LOQ	<LOQ	<LOQ

Table S1.3. Concentrations of organic compounds in the sediments of the twelve studied ponds. “1” and “2” represent 2013 and 2014, respectively.

	Indeno(1,2,3-c,d)pyrene (mg/kg dw)	Chrysene (mg/kg dw)	Naphthalene (mg/kg dw)	Pyrene (mg/kg dw)
sku1	0.04	0.06	0.03	0.34
sku2	<LOQ	<LOQ	<LOQ	0.56
tan1	0.12	0.18	0.09	1.1
tan2	<LOQ	<LOQ	<LOQ	0.61
tak1	0.18	0.13	<LOQ	1.8
tak2	<LOQ	<LOQ	<LOQ	0.67
tas1	0.13	0.12	0.11	0.77
tas2	0.09	0.09	<LOQ	0.23
nøs1	<LOQ	0.06	<LOQ	0.36
nøs2	0.15	0.17	<LOQ	1
vas1	0.18	0.24	<LOQ	1.8
vas2	<LOQ	0.2	<LOQ	1.4
nor1	0.03	0.03	<LOQ	0.15
nor2	<LOQ	<LOQ	<LOQ	0.12
ene1	0.02	<LOQ	<LOQ	0.07
ene2	0.02	<LOQ	<LOQ	0.05
ten1	<LOQ	<LOQ	<LOQ	0.05
ten2	<LOQ	<LOQ	<LOQ	0.05
for1	0.21	0.13	0.07	0.55
for2	<LOQ	<LOQ	<LOQ	1.3
hov1	<LOQ	<LOQ	<LOQ	<LOQ
hov2	<LOQ	<LOQ	<LOQ	0.12
els1	<LOQ	<LOQ	<LOQ	0.07
els2	<LOQ	<LOQ	<LOQ	<LOQ

Table S2. Concentrations of pollutants in the sediments of the twelve studied ponds. “1” and “2” represent 2013 and 2014, respectively. The concentrations that were categorized into class 5, which indicates poor quality that can result in acute toxicity, were marked in bold.

Pond	Pyrene	Cr	Ni	Pb	Cu	Zn	Fe	Al	Ca	Total hydrocarbons	TOC
						mg/kg dw					m-%
sku1	0.34	44	31	23	110	420	32000	16000	8200	2850	4.2
sku2	0.56	73	49	41	**190**	720	40000	23000	7300	1800	7
tan1	1.1	51	35	27	**200**	690	37000	18000	9600	5810	13
tan2	0.61	41	32	23	**160**	530	30000	14000	6300	3200	9.5
tak1	1.8	49	53	34	**190**	800	61000	20000	11000	32000	12
tak2	0.67	48	61	35	**200**	730	60000	21000	10000	6590	8.3
tas1	0.77	61	45	34	**170**	850	37000	23000	9700	3170	12
tas2	0.23	48	52	35	88	420	34000	22000	5100	380	6.3
nøs1	0.36	41	39	21	65	290	38000	22000	13000	4170	3.4
nøs2	1	48	37	22	**150**	720	31000	16000	10000	4100	8.3
vas1	1.8	42	32	21	130	740	38000	19000	20000	11830	10
vas2	1.4	43	35	17	140	740	34000	16000	17000	6000	11
nor1	0.15	28	24	25	60	370	24000	15000	26000	1510	7
nor2	0.12	37	32	34	88	430	35000	18000	7600	530	11
ene1	0.07	22	17	18	33	300	19000	10000	5000	987	4.7
ene2	0.05	20	17	20	31	210	19000	9300	4100	220	5.8
ten1	0.05	32	34	15	41	150	28000	15000	11000	679	1.7
ten2	0.05	31	38	14	50	210	26000	12000	4700	310	1.9
for1	0.55	86	110	44	120	510	45000	23000	7300	2740	7.2
for2	1.3	76	81	28	**170**	550	39000	23000	30000	3700	7.2
hov1	0	38	76	76	130	620	58000	17000	7400	380	6.4
hov2	0.12	50	85	69	**190**	560	64000	24000	6300	450	3.7
els1	0.07	25	39	36	64	260	32000	11000	4700	320	3.3
els2	0	15	21	9.9	18	57	15000	6800	1000	<LOQ	0.83

Table S3. Abbreviations of macroinvertebrates shown in Figure 3.

Abbreviation	Taxa name
CloSim	*Cloeon simile*
Gyrini	Gyrinidae
GySpLr	*Gyrinus* sp., larver
EphVul	*Ephemera vulgata*
CaeHor	*Caenis horaria*
LepMar	*Leptophlebia marginata*
LepVes	*Leptophlebia vespertina*
EphDan	*Ephemera danica*
CallSp	*Callicorixa* sp.
SigaSp	*Sigara* sp.
NotGla	*Notonecta glauca*
CloIns	*Cloeon inscriptum*
LumVar	*Lumbriculus variegatus*
LimRho	*Limnephilus rhombicus*
HeSpIm	*Helophorus* sp., imago
ZoniSp	*Zonitoides* sp.
LymPal	*Lymnaea palustris*
Syrphi	Syrphidae
RadBal	*Radix balthica*
TanInd	Tanypodinae indet
PeriSp	*Pericoma* sp.
AseAqu	*Asellus aquaticus*
GyrAlb	*Gyraulus albus*
BaeRho	*Baetis rhodani*
Chiron	Chironomidae

Table S4. Abbreviations of macroinvertebrates and zooplankton shown in Figure 4.

Macroinvertebrates		Zooplankton	
Abbreviation	Species name	Abbreviation	Species name
Halipld	Haliplidae	CyclStrn	*Cyclops strenuus*
CoenHast	*Coenagrion hastulatum*	CyprOpht	*Cypria ophthalmica*
ChirSp	*Chironomus* sp.	AcanVern	*Acanthocyclops vernalis/robustus*
LumbVari	*Lumbriculus variegatus*	SimcExpn	*Simocephalus expinosus*
DixelSp	*Dixella* sp.	DiacBics	*Diacyclops bicuspidatus*
ChaoCrys	*Chaoborus crystallinus*	NotdMonc	*Notodromas monacha*
LymnPals	*Lymnaea palustris*	MacrAlbd	*Macrocyclops albidus*
ChaoObsc	*Chaoborus obscuripes*	SimcVetl	*Simocephalus vetulus*
CorxLarv	Corixidae, larver	ChydSpha	*Chydorus sphaericus*
Ceratopg	Ceratopogonidae	CandCand	*Candona candida*
Dytiscd	Dytiscidae	DaphPulx	*Daphnia pulex*
HolcDubi	*Holocentropus dubius*	CyprVidu	*Cypridopsis vidua*
CloeSp	*Cloeon* sp.	EucyLill	*Eucyclops lilljeborgi*
DiptIndt	Diptera Indet	MacrFusc	*Macrocyclops fuscus*
Acari	Acari	MegcVird	*Megacyclops viridis*
CloeInsc	*Cloeon inscriptum*	OstrcSpp	*Ostracoda* spp.
TanpIndt	Tanypodinae Indet	DapLonSS	Daphnia longispina s.str.
Gerrida	Gerridae	MegcGigs	*Megacyclops gigas*
BaetIndt	Baetidae Indet	KertQuad	*Keratella quadrata*
LeptVesp	*Leptophlebia vespertina*	EucyMacr	*Eucyclops macruroides*

Table S5. Abbreviations of macroinvertebrates and plants shown in Figure 5A-B.

Macroinvertebrates		Plants on the edge of the ponds	
Abbreviation	Species name	Abbreviation	Species name
HolcDubi	*Holocentropus dubius*	CarxVesc	*Carex vesicaria*
ChaoObsc	*Chaoborus obscuripes*	ScutGalr	*Scutellaria galericulata*
CoenHast	*Coenagrion hastulatum*	IrisPseu	*Iris pseudacorus*
ChaoCrys	*Chaoborus crystallinus*	CarxAcut	*Carex acuta*
Halipld	Haliplidae	LythSalc	*Lythrum salicaria*
LumbVart	*Lumbriculus variegatus*	TyphLatf	*Typha latifolia*
BaetIndt	Baetidae Indet	ScirSylv	*Scirpus sylvaticus*
ChirSp	*Chironomus* sp.	LysmVulg	*Lysimachia vulgaris*
DixelSp	*Dixella* sp.	AlisPlan	*Alisma plantago-aquatica*
LymnPals	*Lymnaea palustris*	PhrgAust	*Phragmites australis*
Dytiscd	Dytiscidae	CalmCans	*Calamagrostis canescens*
Gerrida	Gerridae	PhalArun	*Phalaroides arundinacea*
CloeInsc	*Cloeon inscriptum*	LycpEurp	*Lycopus europaeus*
TanpIndt	Tanypodinae Indet	JuncEffs	*Juncus effusus*
CloeSp	*Cloeon* sp.	GlycFlui	*Glyceria fluitans*
DiptIndt	Diptera Indet	AgrsStol	*Agrostis stolonifera*
Acari	Acari	CarxRost	*Carex rostrata*
CorxLarv	Corixidae, larver	EplCilSs	*Epilobium ciliatum ssp. ciliatum*
Ceratopg	Ceratopogonidae	DesCesSs	*Deschampsia cespitosa ssp. cespitosa*
LeptVesp	*Leptophlebia vespertina*	JuncBufn	*Juncus bufonius*

Table S6. Abbreviations of macroinvertebrates and plants shown in Figure 5C-D.

Macroinvertebrates		Plants within the ponds	
Abbreviation	Species name	Abbreviation	Species name
ChirSp	*Chironomus* sp. (store)	ButmUmbl	*Butomus umbellatus*
BaetIndt	Baetidae indet	PersAmph	*Persicaria amphibia*
DixelSp	*Dixella* sp.	PhrgAust	*Phragmites australis*
Dytiscd	Dytiscidae	Poaceae	Poaceae
CloeInsc	*Cloeon Inscriptum*	GlycFlui	*Glyceria fluitans*
CloeSp	*Cloeon* sp.	AlsPlnAq	*Alisma plantago aquatica*
Gerrida	Gerridae	SparErec	*Sparganium erectum*
Ceratopg	Ceratopogonidae	SchoLacs	*Schoenoplectus lacustris*
CoenHast	*Coenagrion hastulatum*	TyphLatf	*Typha latifolia*
HolcDubi	*Holocentropus dubius*	PotmBerc	*Potamogeton berchtoldii*
Acari	Acari	SparAngs	*Sparganium angustifolium*
TanpIndt	Tanypodinae Indet	LemnMinr	*Lemna minor*
CorxLarv	Corixidae, larver	PotmNatn	*Potamogeton natans*
LumbVari	*Lumbriculus variegatus*	JuncBulb	*Juncus bulbosus*
RadxBalt	*Radix baltica*	PotmAlpn	*Potamogeton alpinus*
LymnPals	*Lymnaea palustris*	UtrcVulg	*Utricularia vulgaris*
NotnLarv	Notonectidae, larver	NuphLute	*Nuphar lutea*
ChaoCrys	*Chaoborus crystallinus*	UtrcVulg	*Utricularia vulgaris*
ChaoObsc	*Chaoborus obscuripes*	ElodCand	*Elodea canadensis*
DiptIndt	Diptera Indet	CalltSp	*Callitriche* sp.
		ComrPals	*Comarum palustre*

Table S7. The Norwegian River Basin Specific Pollutants Environmental Quality Standards (EQS) for chromium, nickel, lead, copper, zinc, and pyrene in the sediments (Vannportalen, 2018).

Substance	Unit	Class I Background	Class II AA-EQS[a]	Class III MAC-EQS[b]	Class IV	Class V Comprehensive acute tox eff.
Pb	mg/kg TS	25	150	1480	2000	2000-2500
Ni	mg/kg TS	30	42	271	533	> 533
Cu	mg/kg TS	20	84	84	147	> 147
Zn	mg/kg TS	90	139	750	6690	> 6690
Cr	mg/kg TS	60	660	6000	15500	15500–25000
Pyrene	µg/kg TS	5.2	84	840	8400	> 8400

[a] The EQS is expressed as an annual average value (AA-EQS).

[b] The EQS is expressed as a maximum allowable concentration (MAC-EQS).

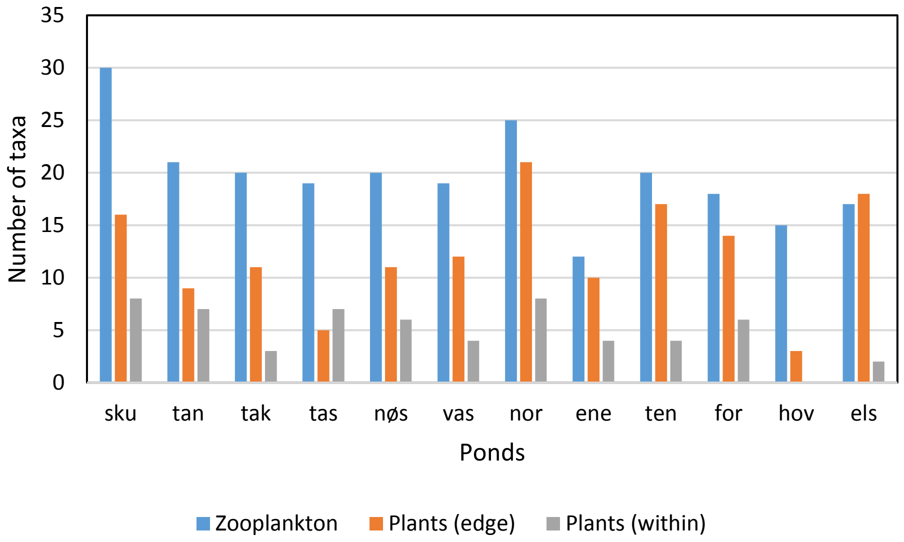

Figure S1. Total number of zooplankton as well as plants within and along the edge of the ponds in the twelve studied ponds in 2013. The following abbreviations are used for the twelve studied ponds: SKU - Skullerud, TAN – Taraldrud North, TAK – Taraldrud crossing, TAS – Taraldrud south, NØS – Nøstvedt, VAS – Vassum, NOR – Nordby, ENE – Enebekk, ELS – Elstadmoen, HOV – Hovinmoen, FOR – Fornebu, TEN – Tenor.

References

VANNPORTALEN 2018. Klassifisering av miljøtilstand i vann - Økologisk og kjemisk klassifiseringssystem for kystvann, grunnvann, innsjøer og elver. Vannportalen.
